# PPARs in Liver Diseases and Cancer: Epigenetic Regulation by MicroRNAs

**DOI:** 10.1155/2012/757803

**Published:** 2012-09-13

**Authors:** Marion Peyrou, Pierluigi Ramadori, Lucie Bourgoin, Michelangelo Foti

**Affiliations:** Department of Cell Physiology and Metabolism, Faculty of Medicine, Centre Médical Universiatire (CMU), 1206 Geneva, Switzerland

## Abstract

Peroxisome-proliferator-activated receptors (PPARs) are ligand-activated nuclear receptors that exert in the liver a transcriptional activity regulating a whole spectrum of physiological functions, including cholesterol and bile acid homeostasis, lipid/glucose metabolism, inflammatory responses, regenerative mechanisms, and cell differentiation/proliferation. Dysregulations of the expression, or activity, of specific PPAR isoforms in the liver are therefore believed to represent critical mechanisms contributing to the development of hepatic metabolic diseases, disorders induced by hepatic viral infections, and hepatocellular adenoma and carcinoma. In this regard, specific PPAR agonists have proven to be useful to treat these metabolic diseases, but for cancer therapies, the use of PPAR agonists is still debated. Interestingly, in addition to previously described mechanisms regulating PPARs expression and activity, microRNAs are emerging as new important regulators of PPAR expression and activity in pathophysiological conditions and therefore may represent future therapeutic targets to treat hepatic metabolic disorders and cancers. Here, we reviewed the current knowledge about the general roles of the different PPAR isoforms in common chronic metabolic and infectious liver diseases, as well as in the development of hepatic cancers. Recent works highlighting the regulation of PPARs by microRNAs in both physiological and pathological situations with a focus on the liver are also discussed.

## 1. Introduction

Obesity, the metabolic syndrome (*MetS*), diabetes, hepatitis viruses (*HBV/HCV*), and abusive alcohol consumption are currently the principal etiological factors favoring the occurrence of hepatocellular adenoma and carcinoma worldwide [1–8]. With obesity, MetS, and diabetes, the liver can develop a wide spectrum of disorders, occurring in individuals in absence of excessive alcohol consumption, or HBV/HCV infections, and ranging from insulin resistance (*IR*), nonalcoholic fatty liver diseases (*NAFLD, including steatosis and insulin resistance*), to nonalcoholic steatohepatitis (*NASH*, *inflammation, and fibrosis associated with steatosis*), and to cirrhosis (*characterized by replacement of liver tissue by extracellular matrix and regenerative nodules*) [9, 10]. Hepatocellular adenoma (*HCA*) and carcinoma (*HCC*) can then occur as the ultimate stage of these diseases [11–13]. Since obesity and metabolic diseases have reached pandemic proportions worldwide, the incidence of IR/NAFLD/NASH/cirrhosis and HCA/HCC is expected to dramatically increase in the future, likely becoming the most common hepatic diseases worldwide [1, 6]. With hepatitis viruses (*HBV/HCV*) and abusive alcohol consumption, a very similar spectrum of histological abnormalities is observed in the liver (*ranging from IR to hepatic steatosis, steatohepatitis, fibrosis, and cirrhosis*) and also precedes HCA/HCC development. However, the molecular mechanisms triggering IR, steatosis, inflammation, fibrosis and cirrhosis are significantly different depending on the etiologies of these hepatic diseases.

Hepatic steatosis consists in the excessive accumulation of neutral lipids (*mainly triglycerides and cholesterol esters*) in cytoplasmic lipid droplets of the hepatocytes. This abnormal accumulation of cytoplasmic lipid droplets can result from (i) an excessive importation of free fatty acids (*FFAs*) released by adipocytes, or coming from the diet; (ii) from a diminished hepatic export of FFA (*altered synthesis or secretion of VLDL*); (iii) an increased *de novo* lipogenesis; (iv) an impaired *β*-oxidation of FFA [14, 15]. Steatosis and hepatic IR are closely associated but it is still poorly understood whether it is steatosis, which causes IR, or *vice versa*. It is clear however that steatosis and IR usually precede inflammation, fibrosis, and cirrhosis of the liver. Recent evidence indicates that progression toward inflammation, fibrosis, and cirrhosis is likely due to the appearance of additional multiple dysregulated mechanisms, including the production of lipotoxic intermediates, oxidative stress (e.g., *through alterations of the mitochondrial activity or lipid peroxidation*), mitochondrial/peroxisomal abnormality, altered nuclear receptors signaling, deregulated cytokines signalling, gut microbial signalling, hepatocyte apoptosis, and leptin resistance, all of them contributing to various extent to the progression towards inflammation and fibrosis/cirrhosis [14–18]. These are thus multifactorial diseases, for which the precise orchestration of their development and progression still remains unclear. In addition, since not all patients with steatosis develop substantial liver injuries, this varying susceptibility of individuals to progress towards inflammation/fibrosis and cirrhosis further indicates that these disorders involve multifaceted processes also highly dependent on environmental factors and genetic predisposition [16–18]. 

Steatosis, inflammation, fibrosis, and cirrhosis can also be regarded as preneoplastic states of the liver, since HCA/HCC might occur as an end stage of these diseases [13]. HCC is a cancer with a very poor prognosis and its clinical management has been the object of intensive research efforts. Surgical resection of the tumour and liver transplantation are currently the only treatments with curative potential. However, only few patients are eligible for surgery and critical issues are associated with tumour resection and the efficiency of hepatic regeneration. Recent evidence indicates that physical inactivity and fat-enriched diets, both becoming major health emergencies in our western society, have a significant impact not only on HCC occurrence and progression, but also on the liver regeneration process. Based on these findings, a major challenge today is to understand how specific nutrients, in particular fats, and an abnormal hepatic lipid metabolism, affect major signalling pathways promoting carcinogenesis.

Peroxisome-proliferator-activated receptors (PPARs) are ligand-activated nuclear receptors that exert a transcriptional activity regulating energy homeostasis and other basic cellular processes in multiple metabolically active organs. These receptors are classically ligand activated, with the best characterized ligands being fatty acids and their derivatives. Three major isoforms of PPARs (*PPAR*α*, PPAR*β*/*δ*, and PPAR*γ**) have been characterized to date [19]. Specific isoforms of these PPARs are expressed in most of the highly metabolically active tissues such as skeletal muscles, heart, adipose tissue, and liver. In the liver specifically, PPARs regulate a whole spectrum of physiological functions including cholesterol and bile acid homeostasis, lipid and glucose metabolism, inflammatory responses, regenerative mechanisms, cell differentiation, and cell cycle [20, 21]. Dysregulations of the expression, or activity, of specific PPAR isoforms are now also well accepted to represent critical mechanisms contributing to the development of a wide range of liver diseases. Indeed, PPARs have been recently implicated in the development of widely spread diseases affecting the liver such as IR, NAFLD/NASH, alcoholic liver diseases (*ALD*), HBV/HCV infection, and HCA/HCC. 

## 2. Role of Specific PPAR Isoforms in Liver ****Diseases

### 2.1. PPARs in Hepatic Insulin Resistance and Steatosis

The role of specific PPARs isoforms in the occurrence and development of hepatic IR and steatosis is still controversial, as well as the potential benefits of specific PPARs agonists to treat these diseases [22]. It is however well accepted that PPARs are tightly implicated in processes regulating the accumulation and storage of triglycerides, lipid oxidation, and insulin sensitivity of hepatocytes [23]. 

#### 2.1.1. PPAR*γ*

PPAR*γ* is generally increased in steatotic livers of both animal models of obesity and human obese patients [24, 25]. *In vivo* studies demonstrated that hepatocytes- and macrophage-specific PPAR*γ* knockout mice were protected against high-fat (*HF*) diet-induced steatosis development [26]. As well, knockdown of PPAR*γ* using *in vivo* injection of adenoviruses could also improve fatty liver and inflammation in mice fed a high saturated fat diet [27]. However, *in vitro* or *in vivo* studies with PPAR*γ* agonists have led to contradictory results regarding steatosis and IR development. For example, thiazolidinediones induce steatosis in human primary hepatocytes and hepatoma cells [28], but reduce *in vivo* hepatic steatosis potentially through upregulation of adiponectin production and adiponectin receptors expression in the liver and adipose tissue [29–32]. On the other hand, SKLB102 (*a barbituric acid-based derivative PPAR*γ* agonist*) was shown to activate lipogenesis and to exacerbate steatosis in mice fed a high-fat/high-calorie diet, although this agonist appears to improve the general outcome of NAFLD/NASH, likely by stimulating insulin sensitivity in both mice or hepatoma cells [30]. In human several clinical studies have reported that thiazolidinediones (rosiglitazone and pioglitazone) lead to a decreased hepatic steatosis and a regression of lobular inflammation [33–35]. It is worthy to note that in human, genetic variants of PPAR*γ* (*C161T, Pro12Ala*) or dominant-negative mutations are specifically associated with NAFLD and progression of NAFLD towards inflammation and fibrosis [36–41]. 

#### 2.1.2. PPAR*α*

As opposed to PPAR*γ*, the role of PPAR*α* to prevent hepatic steatosis, likely by favoring fatty acid oxidation, is supported by a number of studies. Indeed, PPAR*α* expression is decreased in the liver of rodents with NAFLD [42] and PPAR*α* knockout mice display an increased steatosis, oxidative stress, and inflammation when fed an HF Western diet [43, 44]. Fenofibrates, a class of PPAR*α* agonists, were also shown to improve hepatic steatosis in a mouse model of hereditary fatty liver in absence of obesity or diabetes [45] and in OLETF rats, which spontaneously develop NAFLD [32]. Other PPAR*α* agonists (e.g.,* Wy 14 643*) administered to mice fed an HF diet failed to prevent liver inflammation, but could improve other metabolic parameters such as hyperglycemia and hepatic steatosis [46]. Interestingly, the protective effects of fish oil (*n-3 polyunsaturated fatty acids*) dietary supplementation in mice fed a choline/methionine-deficient diet, or of aldose reductase inhibition in the diabetic db/db mouse model, on steatosis development were strongly correlated with a significant increase in the expression of PPAR*α* [47, 48]. Finally, as for PPAR*γ*, a human genetic variant of PPAR*α* (*Val227Ala*) was specifically associated with NAFLD [49]. Although PPAR*α* agonists are used in clinic to treat mixed dyslipidemia and hypertriglyceridemia, few studies have investigated the outcomes of these treatments for NAFLD. Fenofibrate in conjunction with dietary guidelines led to an improved steatosis and liver enzymes in 42% of patients included in the study (*n* = 62) [50]. On the contrary, in another pilot study by Fernandez-Miranda et al., administration of fenofibrate could partially improve liver enzyme profiles and hepatocellular ballooning in patients with biopsies-confirmed NAFLD, but not steatosis, inflammation, and fibrosis [51]. Therefore, from the current available data, the therapeutic potential of PPAR*α* agonists to treat IR and NAFLD in human remains to be evaluated. 

#### 2.1.3. PPAR*β*/*δ*

Recent evidence indicates that PPAR*β*/*δ* can exert a protecting activity against IR. *In vitro* studies showed that PPAR*β*/*δ* expression in HepG2 hepatoma cells could decrease steatosis and IR induced by oleic acid likely by up-regulating PTEN expression and activity [52]. Consistent with these data, PPAR*β*/*δ* knockout mice develop glucose intolerance in part by decreasing the hepatic carbohydrate catabolism [53], but PPAR*β*/*δ*-deficient mice seem to have no defect in liver triglyceride and glycogen accumulation [54]. Whether PPAR*β*/*δ* positively or negatively regulates lipid metabolism and steatosis development remains to date still controversial. Indeed, expression of PPAR*β*/*δ* in the liver of mice through adenoviral infection led to either amelioration of hepatic steatosis in obese db/db mice [55] or exacerbated accumulation of lipids in hepatocytes from HF-fed mice [56]. Several other studies with specific agonists of PPAR*β*/*δ* reported also a beneficial effect of this nuclear receptor activation on hepatic IR and steatosis development. For example, the PPAR*β*/*δ* agonist GW501516 could prevent cytokine-induced IR in human hepatoma cells and in mice liver cells [57] or could improve IR in db/db mice by suppressing hepatic glucose output [53]. GW501516 also alleviated liver steatosis by increasing fatty acid oxidation in mice fed an HF diet [58] or in mice fed a methionine/choline-deficient diet [59]. Another agonist, NNC61-5920, was able to attenuate hepatic IR and to improve the expression profile of genes involved in the lipid metabolism both in mice and rats fed an HF diet. However, whereas systemic insulin sensitivity was improved in mice fed a HF diet, rats under the same conditions displayed systemic IR, suggesting that PPAR*β*/*δ* agonists induced specie-specific metabolic effects [60]. Pharmacological activation of PPAR*β*/*δ* by L-165041 in mice rendered obese and diabetic by HF feeding could also decrease IR and hepatic steatosis [61], whereas steatosis and inflammation were improved in LDLR−/− mice fed a Western diet and treated with L-165041 [62]. Clinical trials with PPAR*β*/*δ* agonists have recently provided also encouraging perspectives to treat NAFLD. Indeed, healthy volunteers treated with GW501516 displayed reduced plasmatic triglycerides after two weeks [63], whereas, in overweighted subjects, GW501516 led to reduced plasmatic triglycerides and LDL levels and a decreased fat content in the liver [64]. Finally, MBX-8025, a novel PPAR*β*/*δ* agonist, decreases liver enzymes, hypercholesterolemia and hypertriglyceridemia in dyslipidemic overweighted patients [65].

### 2.2. PPARs in Liver Inflammation and Fibrosis

Evidence indicates that defects in signalling associated with all three isoforms of PPARs could further contribute to the progression of hepatic IR and steatosis towards more severe stages of liver diseases.

#### 2.2.1. PPAR*γ*

Several studies indicated that PPAR*γ* deficiency in hepatic stellate cells is associated with a transdifferentiation and activation of these cells leading to excessive formation of fibrotic tissue in the liver [66–68]. Surprisingly however, a recent study by Yamazaki and colleagues concluded that downregulation of PPAR*γ*2 in the liver through adenovirus injection improves inflammation and steatosis in mice fed a high-saturated-fat diet [27]. Available data with PPAR*γ* agonists consistently suggest that activation of PPAR*γ* signalling protects the liver against fibrosis and inflammation in mice and rats. Indeed administration of rosiglitazone to mice or rats fed a choline/methionine-deficient diet prevented fibrosis development [69, 70]. In another study, rosiglitazone was also reported to stimulate antioxidant gene expression, *β*-oxidation of fatty acids and to suppress inflammation and fibrosis in mouse models of NASH [31]. Finally, in human with NASH, pioglitazone significantly improved the biochemical and histological feature of NASH and discontinuation of the treatment led to a rebound of the diseases [71].

#### 2.2.2. PPAR*α*

Consistent with a protective role of PPAR*α* for IR and steatosis development, PPAR*α*-null mice fed a Western diet develop more steatosis, oxidative stress, and inflammation in the liver than wild-type mice [44]. Deletion of PPAR*α* in apoE2-KI mice fed a Western diet also aggravates liver steatosis and inflammation development. However, agonists of PPAR*α* administered to foz/foz mice (*rendered diabetic by feeding with a HF diet*) failed to resolve liver inflammation although other histological parameters, including steatosis, hepatocytes ballooning and neutrophils/macrophages recruitment, were improved [46]. Intriguingly, the beneficial effects of vitamin E on NASH in human were correlated with a decreased, and not an increased, expression of PPAR*α* [72].

#### 2.2.3. PPAR*β*/*δ*

PPAR*β*/*δ*-deficient mice appear to be more sensitive to chemical hepatotoxic agents. For example, PPAR*β*/*δ* knockout mice treated with CCL_4_ developed more liver necrosis, displayed more elevated ALT enzymes, inflammation and profibrotic genes expression than control mice, therefore suggesting that PPAR*β*/*δ* protects the liver from inflammation and fibrosis [73]. Consistent with this study, transcriptional genomic analysis with the liver of PPAR*β*/*δ*-deficient mice suggested that PPAR*β*/*δ* could have an anti-inflammatory action in the liver [54] and the PPAR*β*/*δ* agonist GW501516 was shown to improve hepatic inflammation in mice fed a methionine/choline-deficient diet (*a widely used model for NASH*) [59].

Interestingly, PPAR*β*/*δ* seems to be required in Kupffer cells to support the switch between the proinflammatory macrophage M1 to the anti-inflammatory macrophage M2 [74], whereas in stellate cells PPAR*β*/*δ* is highly up-regulated following activation [75]. It is thus likely that PPAR*β*/*δ* has distinct roles in parenchymal and nonparenchymal hepatic cells, which affect, in a still poorly understood manner, the paracrine dialog between these cells and the development of inflammation and fibrosis.

### 2.3. PPARs in Alcoholic Liver Diseases and Hepatitis Virus Infections

Although studies focusing on the role of PPARs in alcoholic liver diseases (ALD) and HBV/HCV infections might be sometimes scarce (e.g.,* regarding the role of PPAR*β*/*δ* in these diseases*), evidence accumulates suggesting that an unbalanced expression/activation of distinct PPAR isoforms may also contribute to the wide spectrum of liver disorders induced either by excessive alcohol consumption or HBV/HCV.

#### 2.3.1. PPAR*γ*

In the liver of HCV-infected patients, PPAR*γ* expression was found to be highly up-regulated and to contribute to the development of HCV-associated steatosis [25, 76, 77]. Two viral factors, the core protein and the NS5A protein, were suggested to mediate PPAR*γ* upregulation either through transactivation of LXR*α* [76–78] or by inducing SOCS7 expression [79]. PPAR*γ* is also increased in the liver of HBV-infected patients [80], likely through HBV X protein-dependent mechanisms [81, 82]. However, whether PPAR*γ* confers or not a replicative advantage to HBV still remains a controversial issue [83–85]. 

#### 2.3.2. PPAR*α*

With excessive alcohol consumption, PPAR*α* gene transcription is inhibited through still unknown mechanisms [86] and may contribute, through an increased oxidative stress and inflammatory response, to the wide spectrum of liver disorders observed with alcoholism [87]. PPAR*α* was also reported to be down-regulated by HCV in the liver of infected patients [88, 89]. Since PPAR*α* blocks the replication and production of infectious viral particles, its downregulation likely confers a replicative advantage to the virus in spite of the resulting metabolic disorders for the host cells [90, 91].

#### 2.3.3. PPAR*β*/*δ*

Although virtually none is known about the potential role of PPAR*β*/*δ* in HBV/HCV infection, only one study has outlined the potential benefit of PPAR*β*/*δ* activation in liver injuries related to alcohol consumption. Indeed, pharmacological activation of PPAR*β*/*δ* by L-165041 in rats chronically fed with ethanol attenuated the severity of liver injury by diminishing oxidative stress, lipid peroxidation and by restoring hepatic insulin responsiveness [92].

### 2.4. PPARs in Liver Cancer

As previously mentioned, *HCA* and *HCC* represent the ultimate stages of liver diseases induced by obesity (*NAFLD/NASH*), alcohol consumption, or hepatitis viruses (*HBV/HCV*) [11–13]. It is therefore expected that PPARs signalling can either favor, or refrain, carcinogenic processes in the liver. However, data have revealed that PPARs signalling might have a different outcome on carcinogenesis as compared to other metabolic diseases of the liver. 

#### 2.4.1. PPAR*γ*

Although the role of PPAR*γ* in the development of liver metabolic diseases with different etiologies has led in some cases to controversial results (*see previous sections*), there is a general consensus about the fact that PPAR*γ* activity can counteract the occurrence and progression of cancer in the liver. In *in vitro *studies, PPAR*γ* overexpression induced apoptosis in HCC cell lines [93]. On the other hand, PPAR*γ* agonists administration to HCC cell lines promoted cell cycle arrest, apoptosis, and anoikis [94, 95], sensitized the cells to 5-fluorouracil antitumoural activity [96], and inhibited migration of these cells [97]. Interestingly, in addition to prevent HBV replication *in vitro* [85], PPAR*γ* agonists triggered also antineoplastic effects on HBV-associated HCC cells [98]. *In vivo* studies corroborated data obtained *in vitro*. Indeed, PPAR*γ*-null mice display a higher susceptibility to the development of HCC induced by the carcinogen diethylnitrosamine (*DEN*). The administration PPAR*γ* agonists (*rosiglitazone*) also reduced HCC development induced by DEN in rats or by hepatoma cell xenograft in mice [99–101]. Together, these studies indicate that PPAR*γ* may act as a tumour suppressor and that specific agonists could be used in cancer prevention.

#### 2.4.2. PPAR*α*

As opposed to PPAR*γ*, activation of PPAR*α* in the liver leads to carcinogenesis in rodents [102–104]. Indeed, chronic administration of the PPAR*α* agonists Wy-14, 643 or bezafibrate in mice induced HCC with time, an effect that was not observed in PPAR*α*-null mice [102, 105, 106]. Interestingly, the PPAR*α* carcinogenic effect on the liver seems to be age dependent in rodent, suggesting that with ageing the liver become more sensitive to the carcinogenic affects of PPAR*α* transcriptional activity [107]. An aberrant expression of PPAR*α* also contributes to the hepatocarcinogenic events induced by the solvent trichloroethylene (*TCE*) in rodents [108] and PPAR*α* activation was necessary to induce steatosis and HCC in a model of transgenic mice expressing the HCV core protein [109, 110]. Surprisingly, the carcinogenic effects of PPAR*α* agonists were not confirmed in human patients, suggesting species differences in PPAR*α* signalling [103]. Consistent with this study, mice expressing a human PPAR*α* were less susceptible to develop cancer with agonist administration [111, 112]. These differential susceptibilities to PPAR*α*-induced HCC in rodent versus humans could be related to the inhibition by PPAR*α*, in rodents but not in human, of the expression of the let-7c miRNA, which targets the Myc oncogene [102].

#### 2.4.3. PPAR*β*/*δ*

The role of PPAR*β*/*δ* in cancer is currently the subject of intense investigations that have led to controversial hypothesis. Indeed, different studies support a pro-carcinogenic role for PPAR*β*/*δ* as a differentiation and anti-inflammatory factor [104]. Regarding liver carcinogenesis, only few studies have provided insights into the role of PPAR*β*/*δ* in this process. PPAR*β*/*δ* agonists, such as GW501516, were reported to significantly enhance the growth of various hepatoma cells, whereas inhibition of PPAR*β*/*δ* expression by siRNAs had the opposite effect [113]. PPAR*β*/*δ* transcriptional activity was also involved in the IL-6-induced proliferation of cultured chicken hepatocytes [114]. It thus remains to investigate more in detail how PPAR*β*/*δ* activity impacts on liver cancer.

## 3. MicroRNAs

**Figure 1:**
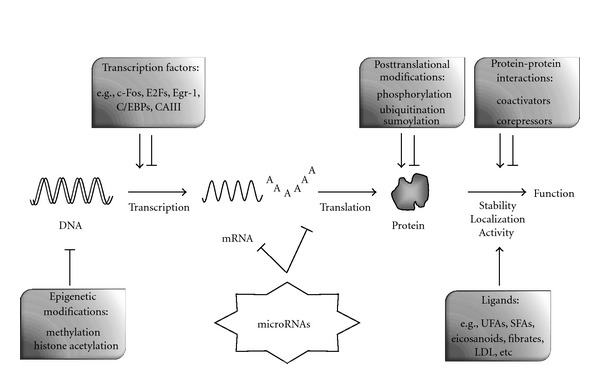
*Regulation of PPARs expression and activity.* The scheme presents distinct mechanisms reported to regulate the expression and activity of specific PPARs isoforms. Epigenetic mechanisms, including DNA methylation and histone acetylation, may restrict the PPARs promoter activity and several transcription factors have been described to modulate either positively or negatively the transcription of distinct PPARs. As well, posttranslational modifications at the protein level (*phosphorylation, ubiquitination, and sumoylation*) and interactions with coactivators (e.g.,* CBP/p300, SRC-1, PGC1*α**) or corepressors (e.g.,* RIP-140*α*, SMRT *α**) regulate the transcriptional activity, localization, and stability of PPARs isoforms. miRNAs represent a new recently described class of regulators of PPARs transcriptional activity by exerting a control on PPARs mRNA degradation and translation.

In addition to PPARs activation triggered by fatty acids and derivatives specific ligands, the transcriptional activity of the various PPARs isoforms in physiological and pathological situations is regulated by other numerous and complex epigenetic, transcriptional, and posttranscriptional mechanisms (Figure 1). These include epigenetic inhibitory mechanisms (e.g.,* methylation of the PPARs promoter and histone acetylation*) [115, 116], positive and negative regulation of PPARs mRNA transcription by various transcription factors [117–120], posttranslational modifications of the proteins, interaction with other cellular factors, which may affect their transcriptional activities, intracellular localization, and stability of the PPARs proteins [19, 121–125]. Interestingly, a further level of complexity in these regulatory mechanisms has recently emerged and involves an epigenetic regulation of PPAR isoforms by microRNAs, which can either degrade or repress PPAR mRNAs at the translational level.

miRNAs are small noncoding RNAs of about 20 nucleotides that bind to conserved 3′UTR sequences of their target mRNA, therefore inducing either inhibition of their translation or their degradation [126]. First described in *C. elegans *[127], 1921 miRNAs have been discovered and registered to date (*according to miRBase microRNA database,* http://www.mirbase.org/) and more than 30% of proteins-coding genes are supposed to be targeted by miRNAs [128]. Most of the miRNAs are intergenics and can be expressed independently, but a few of them are also located in introns of known protein-coding genes and are co-transcribed with these genes [129]. The biogenesis of miRNAs is a well-conserved mechanism. The RNA polymerase II transcribes most of microRNAs as a long primary transcript enclosing a stem-loop structure [130]. This process can be regulated by several transcription factors that bind directly to the miRNA promoter elements and control their expression [131]. miRNAs maturation occurs in the nucleus where the RNase III Drosha and other cofactors cleave the pri-miRNA bearing the hairpin structure to generate a pre-miRNA product [132]. The precursor pre-miRNA of about 60–70 nucleotides is then exported from the nucleus to the cytoplasm by the exportin-5 [133, 134], a process that is regulated by the Drosha activity [135, 136]. In the cytoplasm, the RNase III Dicer cleaves the miRNA stem loop to generate a mature miRNA of 20–22 nucleotides long [137]. Mature miRNA targeting a mRNA finally necessitates the RNA-induced silencing complex RISC, in which the Argonaute protein (*Ago2 for mammals*) is the catalytic center [138, 139]. Of note, Drosha, Dicer, and Ago2 are components of miRNAs processing that are essential for life since specific knockout for one of these proteins in mice induces severe developmental defects or death [140]. 

Finding the targets of specific miRNA currently remains a challenge. Bioinformatic predictions and proteomic analyses are performed to estimate potential targets for a given miRNA [141]. However, there is a great redundancy in miRNAs capable to target a specific mRNA and each miRNA can also target hundreds of different mRNAs [142, 143] thereby increasing tremendously the complexity of this type of global analyses. In addition, miRNAs usually induces only a maximum of twofold downregulation of their target protein, thereby exerting only a fine-tuning control of protein expression. Despite these difficulties to investigate the physiological and pathological potential roles of miRNAs, accumulating evidence indicates that miRNAs play an important role in multiple cellular processes, as well as in the development of several diseases, including the MetS, diabetes, neurodegeneration, cardiovascular and immune diseases [144–148]. Numerous studies also recently outlined miRNAs as critical regulatory factors promoting or inhibiting cancer development [149, 150]. More particularly in the liver, several miRNAs (*including miR-21, miR-29, miR-122, miR-132, miR-155, miR-192, and miR-322*) were reported to exert a control on hepatocyte differentiation, glucose and lipid metabolism, fatty liver diseases, fibrosis, and HBV/HCV infection [151–160]. In liver cancer, for example, HCC, “omics” analyses have revealed that numerous miRNAs are dysregulated. Importantly, while miR-122 is down-regulated in HCC thereby potentially impairing cell differentiation, many others are up-regulated, including miR-96, miR-221, miR-224, and miR-21, which have all been reported to regulate cell proliferation and apoptosis [161, 162].

## 4. MicroRNA-Dependent PPARs Regulation

The epigenetic regulation of PPARs expression and activity by miRNAs is a new field of research still in its infancy. However, accumulating evidence now suggests that alterations of the expression/activity of PPARs isoforms by distinct miRNAs could represent critical molecular mechanisms involved in the physiopathology of each organ undergoing a PPARs-dependent control. In this regard, bioinformatics predictions of miRNAs potentially targeting the different PPARs isoforms reveal that hundreds of different miRNAs could participate to the regulation of PPARs expression/activity in different cells/tissues (see Table 1). Currently, most of the studies investigating the role of miRNAs in the regulation of distinct PPARs isoforms have been performed in cultured adipocytes or in the adipose tissue. But recent works have also highlighted miRNAs-dependent regulation of PPARs in the liver and have implicated these regulatory processes in the development of hepatic diseases.

**MicroRNA-Dependent PPARs Regulation in the Liver**

At least 5 distinct miRNAs have been reported to directly, or indirectly, affect PPAR expression in liver cells, mostly in pathological situations, in particular NAFLD (see Table 2). For example, Zheng and coworkers performed miRNA microarrays with human hepatocyte cell line L02 exposed or not to high concentration of fatty acids. They could find more than 30 miRNA either up- or down-regulated in cells challenged with excess of fatty acids. Among them, miR-10b was up-regulated. Further analyses revealed that miR-10b induces steatosis in these cells by directly targeting PPAR*α* and preventing its expression [163]. PPAR*α* was also reported to be specifically regulated by two other miRNAs, miR-21 and miR-27b, in the liver. Indeed, expression of precursors or antisense nucleotides for miR-21, or miR-27b, in Huh-7 hepatoma cells could significantly modulate the expression of PPAR*α* protein, but not its mRNA, suggesting a blockade of PPAR mRNA translation by miR-21/-27b. Interestingly, no correlations were found between PPAR*α* protein and mRNA expression in human livers (*n* = 24). However, in the same human samples, PPAR*α* protein expression was inversely correlated with miR-21 expression [164]. A potential role for miR-21-dependent regulation of PPARs was further supported by our own observations indicating that PPAR*α* protein expression is up-regulated in primary hepatocytes isolated from miR-21 knockout mice, whereas PPAR*γ* is down-regulated in the liver of mice overexpressing miR-21 (*our unpublished data*). miR-122, the most highly expressed miRNA in the liver, was also reported to specifically target PPAR*β*/*δ* in the liver of mice. Interestingly miR-122 expression appears to be circadian thereby providing an interesting link between miR-122, PPAR*β*/*δ*, and the well-known circadian metabolic control occurring in the liver [165]. Finally, PPAR*γ* activity appears also to be under the indirect control of miR-132. Indeed, Mann and coworkers could demonstrate that, in stellate cells of mice treated with CCl4, downregulation of the miR-132 promoted the expression of MeCP2 (*a target of miR-132*), which in turn binds to PPAR*γ* and promotes the formation of an epigenetic repressor complex inhibiting PPAR*γ* expression and therefore promoting liver fibrosis in this mice model [116].

**MicroRNA-Dependent PPARs Regulation in Other Tissues**

Numerous other reports have implicated an miRNA-dependent regulation of distinct PPARs isoforms in non-hepatic tissues that affects various cellular processes including lipid metabolism, inflammation, cell differentiation, carcinogenesis, or wound skin repair.

In the adipose tissue, two studies have unveiled a physiopathologically relevant regulation of PPAR*γ* expression by the miR-27a/b. Indeed, Karbiener et al. reported a significant downregulation of miR-27b, which directly targets PPAR*γ*, during adipogenesis of human multipotent adipose-derived stem (*hMADS*) cells. They further demonstrated that inhibiting induction of PPAR*γ* in this process by overexpressing miR-27b represses adipogenic marker gene expression and triglyceride accumulation in hMADS [166]. In a second study, levels of miR-27a were inversely correlated with those of PPAR*γ* in mature adipocyte and miR-27a expression in the adipose tissue was down-regulated in obese mice as compared to lean mice. *In vitro* studies with 3T3-L1 preadipocytes further showed that miR-27a ectopic expression prevents adipocyte differentiation by inhibiting PPAR*γ* through a direct binding to its 3′-UTR sequence [167]. Finally, differentiation of human preadipocytes into adipocytes was also shown to be highly dependent of miR-130 expression. Indeed, miR-130 was shown to directly target PPAR*γ* and thereby to enhance adipogenesis. Consistent with this, expression of miR-130 in the adipose tissue of obese women was lower compared to lean women [168]. Together, these studies suggest that induction of PPAR*γ* by alterations in miR-27a/b and/or miR-130 expressions might be linked to the development of obesity in rodents and humans. miRNAs microarray analyses of the subcutaneous adipose tissue in nondiabetic but severely obese adults also identified miR-519d as an overexpressed miRNAs that targets PPAR*α*. The miR-519d-dependent decrease in PPAR*α* translation was further shown to increase lipid accumulation during pre-adipocyte differentiation suggesting that PPAR*α* loss through miR-519d overexpression importantly contributed to adipocytes hypertrophy observed with obesity [169].

Finally, recent evidence also indicates that alterations of PPAR*γ* and PPAR*α* expression by specific miRNAs occur in other tissues and can contribute to the development of specific disorders. For example, downregulation of PPAR*γ* by an aberrant expression of miR-27b in cardiomyocytes was associated with cardiac hypertrophy in mice [170], whereas miR-27b-dependent PPAR*γ* downregulation in macrophages favors the inflammatory response to LPS [171]. In human osteoarthritic chondrocytes, aberrant expression of miR-22 specifically down-regulates PPAR*α* expression, which in turn promotes IL1-dependent inflammatory processes [172]. Then in endothelial cells of cultured human umbilical vein under oscillatory shear stress, levels of miR-21 were found to be increased and to induce an inhibition of PPAR*α* translation, which favor monocytes adhesion and atherosclerosis formation [173]. Finally, PPAR*α* was reported to be also a target of miR-506, which is increased in human colon cancers and contribute to chemotherapy resistance by down-regulating PPAR*α* expression [174].

## 5. Conclusion

Although the therapeutic potential of PPAR*β*/*δ* for metabolic diseases such as insulin resistance, dyslipidemia, and other associated liver pathologies deserves further investigations, agonists of PPAR*γ* and PPAR*α* have been developed as relevant drugs to treat these disorders. For cancer therapies, the use of PPAR agonists is more debated, in particular for PPAR*α*, which induces liver and bladder cancers in mice, but apparently not in humans. However, the chronic administration of PPAR agonists in human for the treatment of metabolic diseases may importantly increase the risk of developing cardiovascular diseases (e.g.,* for PPAR*γ* agonists*) and specific cancers (e.g.,* for PPAR*α* agonists*). For these reasons, recommendations have been issued for a restricted and invigilated clinical use of PPAR agonists and some of them (e.g.,* PPAR*γ* agonists*) have even been withdrawn from the market in some countries. An attractive therapeutic alternative to systemic administration of PPAR agonists to treat metabolic diseases and/or cancers would be to exert more fine tuning of specific PPAR isoforms expressions/activities in the organ of interest, thereby minimizing the deleterious side effects of chronic systemic administration of these agonists. In this regard, miRNAs represent an interesting class of molecules that could be pharmacologically modulated, for example, with antagomirs, to prevent pathological alterations of PPARs expressions and activities. There might be several advantages associated with this type of therapeutic approaches. First, the aberrant expression of specific miRNAs, for example, inhibiting the expression of PPARs in diseases, is often tissue specific. Therefore, the administration of miRNA-targeting drugs should affect principally the injured organs and secondary systemic effects could be minimized as compared to systemic administration of PPAR agonists. Secondly, as mentioned previously, miRNAs can only modestly modulate (*more or less two fold*) the expression of their target mRNAs. However, preventing this pathological miRNA-mediated dysregulations of PPARs expression should help to recover a physiological PPAR transcriptional activity in contrast to the ectopic overactivation of PPARs induced by administration of agonists. Finally, based on bioinformatics predictions (see Table 1), there is a high number of miRNAs predicted to be able to affect the expression of PPARs in pathological situations. It is likely that many other miRNAs would be identified in the future as important PPARs regulators in metabolic diseases and cancer, thereby multiplying the miRNA-based therapeutic targets available to treat these diseases. Based on these considerations, additional studies are now required to further assess the pertinence of miRNA-based therapies to enhance specifically the activity of PPAR isoforms as therapeutic weapons in metabolic diseases and cancer.

## Figures and Tables

**Table:** (a)

PPAR*α*				
miRanda		TargetScan	MicroCosm targets	PicTar
miR-25	miR-7	miR-200	miR-892	miR-19
miR-28	miR-9	miR-203		
miR-32	miR-10	miR-204		
miR-92	miR-17	miR-211		
miR-145	miR-18	miR-214		
miR-155	miR-19	miR-219		
miR-181	miR-20	miR-223		
miR-200	miR-21	miR-291		
miR-216	miR-22	miR-294		
miR-217	miR-23	miR-295		
miR-218	miR-24	miR-302		
miR-224	miR-27	miR-338		
miR-342	miR-34	miR-351		
miR-363	miR-93	miR-372		
miR-367	miR-101	miR-373		
miR-374	miR-105	miR-427		
miR-376	miR-106	miR-428		
miR-421	miR-124	miR-429		
miR-425	miR-125	miR-449		
miR-429	miR-128	miR-506		
miR-431	miR-138	miR-508		
miR-491	miR-141	miR-518		
miR-543	miR-142	miR-519		
miR-590	miR-144	miR-520		
miR-615	miR-150	miR-548		
miR-708	miR-181	miR-590		
miR-873	miR-182	miR-670		
		miR-761		
		miR-1378		
		miR-1420		
		miR-3619		
		miR-4262		
		miR-4319		
		miR-4735		
		miR-4782		
		miR-5127		

**Table:** (b)

PPAR*γ*				
miRanda		Target scan	MicroCosm targets	Pictar
miR-1	miR-328	miR-27	miR-27	miR-27
miR-9	miR-329	miR-128	miR-30	miR-128
miR-18	miR-338	miR-130	miR-33	miR-130
miR-24	miR-340	miR-301	miR-34	miR-301
miR-25	miR-361	miR-454	miR-128	
miR-26	miR-362	miR-721	miR-130	
miR-27	miR-363	miR-3666	miR-142	
miR-32	miR-367	miR-4295	miR-144	
miR-33	miR-370		miR-193	
miR-34	miR-371		miR-301	
miR-92	miR-376		miR-338	
miR-96	miR-411		miR-409	
miR-101	miR-421		miR-431	
miR-122	miR-431		miR-449	
miR-128	miR-448		miR-454	
miR-130	miR-449		miR-513	
miR-133	miR-454		miR-520	
miR-137	miR-485		miR-526	
miR-142	miR-488		miR-545	
miR-144	miR-490		miR-548	
miR-145	miR-505		miR-559	
miR-148	miR-590		miR-574	
miR-150	miR-613			
miR-152	miR-653			
miR-153	miR-758			
miR-181	miR-1271			
miR-182	miR-1297			
miR-185				
miR-194				
miR-199				
miR-204				
miR-206				
miR-211				
miR-224				
miR-301				
miR-324				

**Table:** (c)

PPAR*β*/*δ*			
miRanda	Target scan	MicroCosm targets	Pictar
miR-19	miR-9	miR-17	none
miR-24	miR-17	miR-20	
miR-129	miR-20	miR-24	
miR-133	miR-29	miR-29	
miR-138	miR-34	mi R-33	
miR-140	miR-93	miR-93	
miR-149	miR-106	miR-106	
miR-185	miR-129	miR-136	
miR-196	miR-138	miR-138	
miR-218	miR-148	miR-143	
miR-223	miR-150	miR-149	
miR-326	miR-214	miR-219	
miR-330	miR-223	miR-220	
miR-342	miR-427	miR-222	
miR-487	miR-449	miR-302	
miR-590	miR-518	miR-373	
miR-599	miR-519	miR-378	
miR-653	miR-761	miR-483	
miR-874	miR-3619	miR-492	
	miR-5127	miR-512	
		miR-513	
		miR-519	
		miR-520	
		miR-542	
		miR-544	
		miR-550	
		miR-564	
		miR-576	
		miR-631	
		miR-640	
		miR-657	
		miR-874	
		miR-885	
		miR-921	

**Table 2:** miRNAs reported to regulate PPAR*γ*, PPAR*α*, and PPAR*β*/*δ* expression and activity in the liver.

microRNA	Implication	PPAR targeted	Interaction	Reference
miR-10b	Steatosis	PPAR*α*	Direct	[163]
miR-21	Steatosis	PPAR*α*	Direct	[164]
miR-122	Cholesterol and lipid metabolism	PPAR*β*/*δ*	Direct	[165]
miR-27b	Steatosis	PPAR*α*	Indirect	[164]
miR-132	Fibrosis	PPAR*γ*	Indirect	[116]

## Abbreviations

ALD: Alcoholic liver diseases
FFAs: Free fatty acids
IR: Insulin resistance
HCA: Hepatocellular adenoma
HBV: Hepatitis virus B
HCV: Hepatitis virus C
HCC: Hepatocellular carcinoma
MetS: Metabolic syndrome
PPARs: Peroxisome-proliferator-activated receptors
NAFLD: Nonalcoholic fatty liver disease
NASH: Nonalcoholic steatohepatitis
miRNA: MicroRNA.
